# Monson’s sphere in Chinese young adult females with individual normal occlusion: a preliminary study using digital models

**DOI:** 10.1186/s12903-024-04541-x

**Published:** 2024-08-17

**Authors:** Yajing Wang, Tianqi Wang, Jiangfei Chen, Xiaoni Duan, Dongqiao Liu, Danna Xiao, Chunxiang Zhang

**Affiliations:** 1 Department of Orthodontics, Tianjin Stomatological Hospital, Tianjin, People’s Republic of China; 2 Tianjin Key Laboratory of Oral and Maxillofacial Function Reconstruction, Tianjin Stomatological Hospital, Tianjin, People’s Republic of China; 3 Department of Stomatology, Tianjin Medical University, Tianjin, People’s Republic of China; 4 Department of Prosthodontics, Tianjin Stomatological Hospital, Tianjin, People’s Republic of China; 5 Stomatology Hospital, School of Stomatology, Zhejiang University School of Medicine, Hangzhou, People’s Republic of China; 6 Department of Orthodontics, Zhengzhou Stomatological Hospital, Zhengzhou, Henan People’s Republic of China; 7 Department of Stomatology, Peking University Binhai Hospital, Tianjin, People’s Republic of China

**Keywords:** Individual normal occlusion, Monson’s sphere, Digital models

## Abstract

**Background:**

This study investigated the characteristics of Monson’s sphere in Chinese young adult females with individual normal occlusion to provide a reference for oral rehabilitation in prosthodontic and orthodontic treatments.

**Methods:**

Points at the dental cusps and incisal edges were selected from 51 digital mandibular dental models of Chinese young adult females (aged 18–22 years) with individual normal occlusion. Monson’s spheres were fitted to the selected points based on the least-squares principle and the radii were calculated. The deviation of each selected point from its relative spherical surface was also calculated. The radii and deviations of these points were examined using conventional descriptive statistics and distributions of the most deviated points inside and outside the spheres were analyzed.

**Results:**

The mean radius of Monson’s sphere in Chinese young adult females was 79.60 ± 14.13 mm. The deviation of each selected point from its relative sphere surface was 0.38 ± 0.30 mm. The maximum deviations inside and outside the sphere were 0.93 ± 0.25 mm and 0.95 ± 0.30 mm, respectively. The most deviated points outside the spheres were mainly distributed at the distolingual cusps of the mandibular second permanent molars (31.37%), while those inside the spheres were mainly distributed at the mesiolingual cusps of the mandibular first permanent molars (45.10%).

**Conclusions:**

The radius of Monson’s sphere in Chinese young adult females was smaller than the classic four-inch value suggested by Monson. Deviation was observed from all selected points to their Monson’s sphere surface, with the most deviated points distributed primarily in the molar region.

## Background

The arrangement of teeth in craniofacial structures is characterized by features such as the curve of Spee [1], the curve of Wilson, and Monson’s sphere [2]. These characteristics are important in occlusal reconstruction in dental practice. Monson’s sphere theory correlates tooth arrangement with craniofacial structures and has been applied to dental articulator design, dentition research, and occlusal curve reconstruction [3–5]. In this theory, Monson suggested that the occlusal surface of dentition with a normal jaw is consistent with a sphere with a radius of approximately 4 inches. Monson defined the sphere’s center at the glabella point, with a surface passing bilaterally through the centers of the condyles [2]. Monson’s research was based on Caucasians; however, craniofacial parameters differ among races [6, 7]. Therefore, the value of 4 inches proposed by Monson may not be appropriate for occlusal adjustments or oral rehabilitation in patients of different races.

Moreover, previous studies observed and measured Monson’s sphere from dry skulls [2]. With technological developments, methods such as three-dimensional electromagnetic digitizers, lateral cephalometric films, digital model measurements, and cone-beam computed tomography (CBCT) have been applied [8–13]. However, the results of these studies suggest that Monson’s sphere values may differ across races. While some Chinese researchers have explored Monson’s sphere in the Chinese population [12, 13], these studies simplified the measurement methods or used inconsistent definitions of Monson’s sphere. For instance, Yuelin et al. [12]. used the average distance from the glabella point to the tooth cusps on lateral cephalometric films to represent the radius of Monson’s sphere, ignoring the characteristics of dentition in a three-dimensional space. Wu et al. [13]. obtained the radius of Monson’s sphere by measuring the distance between the midpoint of the condylar anterior surface and the glabella point using CBCT; however, they did not use dentition data, which is an important part of Monson’s theory. Hence, further research on the characteristics of Monson’s sphere in the Chinese population based on three-dimensional dentition data is needed. In recent years, more and more young adult females are seeking orthodontic or prosthodontic treatments to improve dental and facial aesthetics. This study explored the characteristics of Monson’s spheres in Chinese young adult females with individual normal occlusion to provide more information on dentition for prosthodontic and orthodontic treatments.

## Materials and methods

### Ethical considerations

The study was approved by the Medical Ethics Committee of the Tianjin Stomatological Hospital. Informed consent was obtained from all subjects. The samples for this study were collected from Tianjin Medical College students. The sample size was calculated with an alpha value of 0.05 and a power of 80%. Based on the results of previous study conducted by Nam et al. [11] and the sample size calculation formula ($n=\frac{{Z}_{\alpha }^{2}{ \sigma }^{2}}{{\delta }^{2}}$), we calculated that the sample size of females was 42 at least. This study finally included 51 females aged 18–22 years after school health examinations.

### Inclusion and exclusion criteria

The inclusion criteria were: (1) Han Chinese population by origin, aged 18–25 years; (2) complete permanent dentition, except for the third molars; and (3) Class I canine and molar relationship with normal overbite and overjet. The exclusion criteria were: (1) significant tooth wear, large fillings, or restorations; (2) space or crowding of the whole dentition > 2 mm; (3) history of orthodontic or orthognathic treatments; (4) periodontal diseases; (5) symptoms of temporomandibular joint disorder; and (6) systemic diseases.

### Digitization of the dental models and selection of the points

**Fig. 1:**
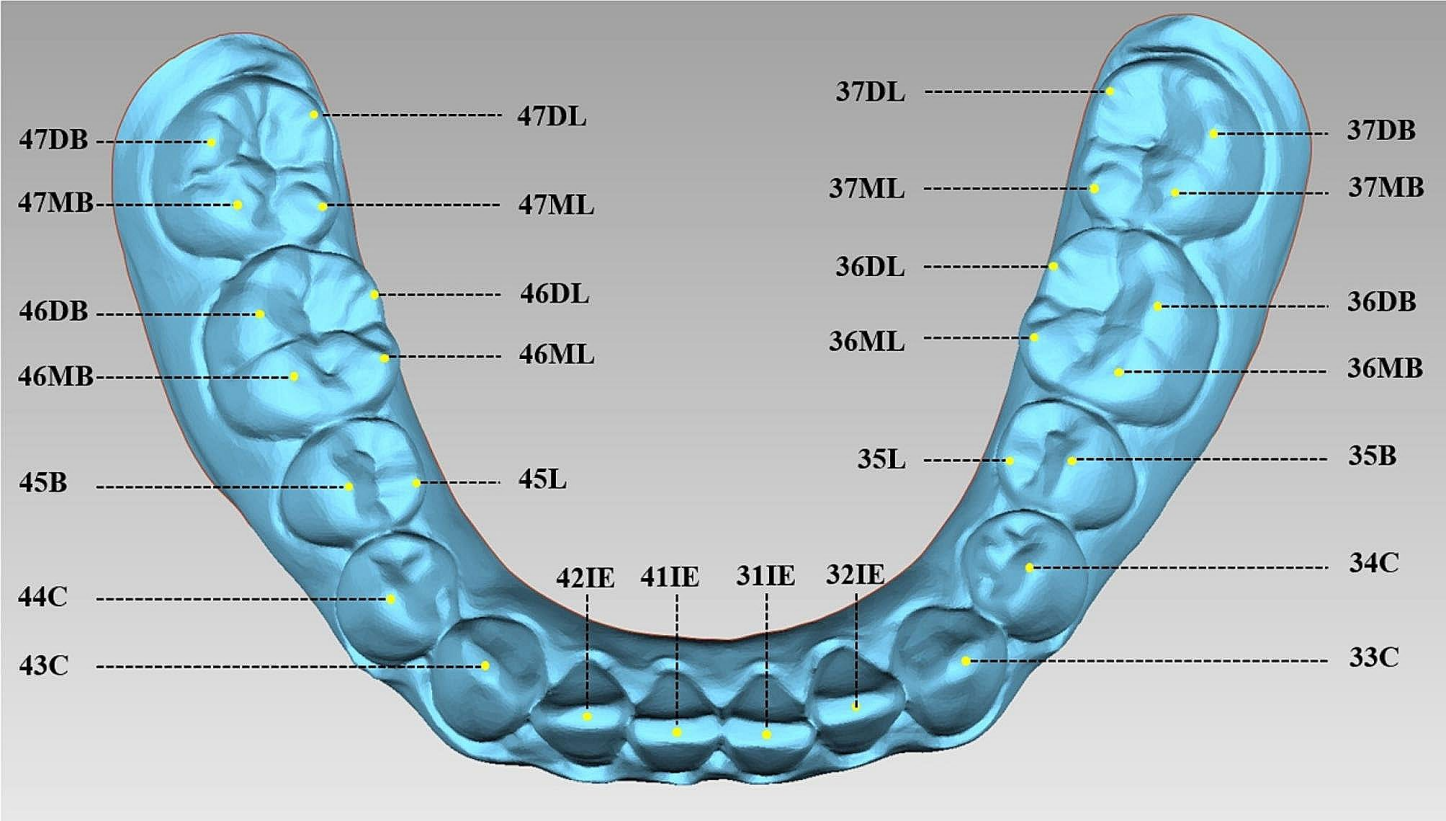
Selected points for sphere fitting

A total of 51 mandibular dental casts were obtained from 51 young adult females aged 18–22 years who met the inclusion criteria. All dental casts were digitized using a model scanner (R700™, 3Shape A/S, Holmens Kanal 7, 1060, Copenhagen, Denmark) and digital model files were saved. As described previously [2, 8, 10, 11], the points selected for fitting the Monson’s sphere in this study were defined and are presented in Table 1; Fig. 1. For several cusps worn into small facets, the center of the facet was selected as the cusp point. All points were selected from digital model files using Geomagic Studio 2012 (Raindrop Geomagic, Research Triangle Park, NC, USA), which output data on their three-dimensional coordinates.

**Table 1:** Definitions of the selected points for fitting Monson’s spheres

Selected points	Definition
1IE	Middle point at incisal edge of the middle incisor
2IE	Middle point at incisal edge of the lateral incisor
3C	Peak point at the cusp of the canine
4C	Peak point at the buccal cusp of the first premolar
5B	Peak point at the buccal cusp of the second premolar
5L	Peak point at the larger one of lingual cusps of the second premolar
6MB	Peak point at the mesiobuccal cusp of the first permanent molar
6ML	Peak point at the mesiolingual cusp of the first permanent molar
6DB	Peak point at the distobuccal cusp of the first permanent molar
6DL	Peak point at the distolingual cusp of the first permanent molar
7MB	Peak point at the mesiobuccal cusp of the second permanent molar
7ML	Peak point at the mesiolingual cusp of the second permanent molar
7DB	Peak point at the distobuccal cusp of the second permanent molar
7DL	Peak point at the distolingual cusp of the second permanent molar

### Monson’s sphere fitting and deviation calculation

All data on the three-dimensional coordinates of the selected points on their relative models were input into MATLAB R2016b (MathWorks Inc., Natick, MA, USA). The radius and center of the Monson’s sphere on each digital model were calculated using a computer program based on the least-squares algorithm. In addition, the deviation of the selected point from the sphere surface was calculated using its sphere radius and the three-dimensional coordinates of the sphere center. The total numbers of most deviated points outside and inside the Monson’s sphere of each digital model were counted.

### Measurement reproducibility

Fifteen digital models were randomly selected to test reproducibility. The points of the 15 digital models for fitting the spheres were selected three times by one investigator at 2-week intervals. The x-, y-, and z-coordinates of each selected point and the calculated radii of their fitting spheres were recorded for error measurements using the intraclass correlation coefficient (ICC). Table 2 presents the ICC results.

**Table 2:** Intraclass correlation coefficients (ICCs) for three calculations

Calculation	ICC
X-coordinate	1.000
Y-coordinate	0.999
Z-coordinate	1.000
Radius	0.999

### Statistical analysis

The collected data were analyzed using IBM SPSS Statistics for Windows, version 23.0 (IBM Corp., Armonk, NY, USA, ). The radii of the Monson’s spheres and deviations of all selected points were examined using conventional descriptive statistics.

## Results

**Fig. 2:**
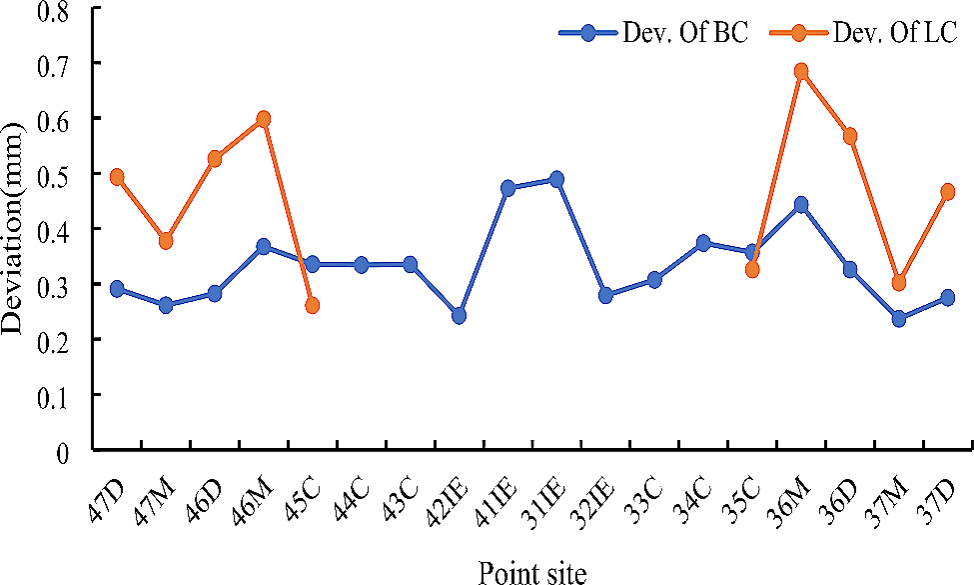
Mean deviations of selected points at each cusp or incisal edge. BC, buccal cusp; LC, lingual cusp

**Fig. 3:**
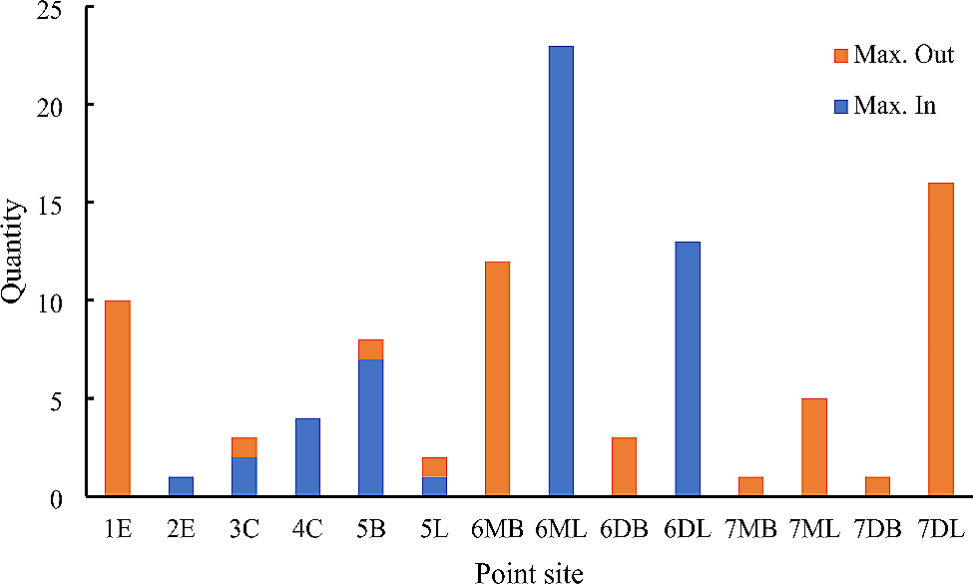
Distribution of most deviated points for each cusp or incisal edge. Max. Out, most deviated points outside the spheres; Max. In, most deviated points inside the spheres

The ICC values shown in Table 2 indicate the excellent reproducibility of the measurements. Overall, the mean Monson’s sphere radius was 79.60 ± 14.13 mm. The mean deviation of all the selected points was 0.38 ± 0.30 mm. The maximum deviations inside and outside the sphere were 0.93 ± 0.25 mm and 0.95 ± 0.30 mm, respectively (Table 3). The mean deviations of the selected points for each cusp and incisal edge are presented in Table 4; Fig. 2. The most deviated points were most commonly observed at the molars. The distributions of the most deviated points on the other sites are shown in Table 5; Fig. 3. Most of the most deviated points inside the sphere were distributed at the lingual cusps of the first permanent molars with 45.10% of those points at the mesiolingual cusps and 25.49% of those points at the distolingual cusps. Most of the most deviated points outside the sphere mainly distributed at the edges of first incisors (19.61%), buccal cusps of first permanent molars (23.53% on the mesiobuccal cusps and 5.88% on the distobuccal cusps) and lingual cusps of second permanent molars (9.80% on the mesiolingual cusps and 31.37% on the distolingual cusps).

**Table 3:** Deviations of selected points

	Total (*n* = 1428)		Max. In (*n* = 51)		Max. Out (*n* = 51)
	(Mean ± SD)		(Mean ± SD)		(Mean ± SD)
Deviation (mm)	0.38 ± 0.30		0.93 ± 0.25		0.95 ± 0.30

Max. In, most deviated points inside the spheres; Max. Out, most deviated points outside the spheres.

**Table 4:** Deviations of selected points at all cusps and incisal edges

Point sites	BC deviation (mm)		LC deviation (mm)
	(Mean ± SD)		(Mean ± SD)
47D	0.29 ± 0.29		0.49 ± 0.34
47M	0.26 ± 0.18		0.38 ± 0.31
46D	0.28 ± 0.22		0.53 ± 0.32
46M	0.37 ± 0.30		0.60 ± 0.35
45C	0.34 ± 0.29		0.26 ± 0.21
44C	0.33 ± 0.30		-
43C	0.34 ± 0.28		-
42IE	0.24 ± 0.20		-
41IE	0.47 ± 0.28		-
31IE	0.49 ± 0.33		-
32IE	0.28 ± 0.22		-
33C	0.31 ± 0.24		-
34C	0.37 ± 0.26		-
35C	0.36 ± 0.26		0.33 ± 0.25
36M	0.44 ± 0.34		0.68 ± 0.34
36D	0.33 ± 0.29		0.57 ± 0.33
37M	0.24 ± 0.19		0.30 ± 0.21
37D	0.28 ± 0.24		0.47 ± 0.41

BC, buccal cusp; LC, lingual cusp; BC deviation: deviation of the selected point at the BC; LC deviation, deviation of the selected point at the LC; D, distal; M, mesial; C, cusp; IE, incisal edge.

**Table 5:** Quantity and percentage of the most deviated points at different point sites

Point sites	Max. In			Max. Out	
	Quantity	Percentage		Quantity	Percentage
1IE	0	0.00%		10	19.61%
2IE	1	1.96%		0	0.00%
3C	2	3.92%		1	1.96%
4C	4	7.84%		0	0.00%
5B	7	13.73%		1	1.96%
5L	1	1.96%		1	1.96%
6MB	0	0.00%		12	23.53%
6ML	23	45.10%		0	0.00%
6DB	0	0.00%		3	5.88%
6DL	13	25.49%		0	0.00%
7MB	0	0.00%		1	1.96%
7ML	0	0.00%		5	9.80%
7DB	0	0.00%		1	1.96%
7DL	0	0.00%		16	31.37%

## Discussion

Monson’s sphere theory characterizes the arrangement of teeth in a three-dimensional space and has been used to reconstruct occlusal curves during oral rehabilitation [4, 14]. This theory has also been used to evaluate occlusal curves in digital models [5, 15]. However, the results of studies on Monson’s sphere are controversial because of differences in research methods and participant age and race. In addition, research on Monson’s spheres in Chinese young adult females with individual normal occlusion is insufficient. Therefore, further investigation of Monson’s spheres in China is needed to provide information for individualized occlusal adjustment. This study took advantage of digital models to analyze the characteristics of Monson’s spheres in Chinese young adult females.

Yuelin et al. [12]. attempted to substitute the Monson’s sphere with the curve of Spee on cephalometric films; however, the results may be inaccurate owing to the distortion of the teeth on the films. Wu et al. [13]. determined the radius of Monson’s sphere by measuring the distance between the midpoint of the condylar anterior surface and the glabella point using cone-beam computed tomography. This method was so simplified that detailed dentition information could be ignored. Ferrario et al. [9]. transformed three-dimensional coordinates into two-dimensional data to calculate the Monson’s sphere radius. Kagaya et al. [10]. calculated the Monson’s sphere radius in Japanese adults by restricting the sphere’s center to one of the coordinate planes. Both these methods sacrificed part of the three-dimensional information of the Monson’s sphere to some extent. Nam et al. [11]. developed a best-fit algorithm to produce a Monson’s sphere using selected points on digital models. This method made full use of the three-dimensional coordinates of the selected points. However, Nam et al. [11]. did not consider the incisors as part of Monson’s sphere; and abandoned the incisors when selecting points that differed from Monson’s theory. As part of the entire dentition, incisal guidance is relevant to mandibular movement and temporomandibular joint development [16, 17]. Thus, it is more reasonable to include incisors when researching Monson’s spheres. The present study adopted a method similar to that of Nam et al. but added points on the incisors. Furthermore, unlike previous studies, this study analyzed the deviation of each selected point from its relative sphere surface. This study included 51 Chinese young adult females with individual normal occlusion (mean age 19.08 ± 1.28 years). The mean Monson’s sphere radius was 79.60 ± 14.13 mm, which was smaller than the original 4-inch value measured by Monson [2]. The dentition of these subjects was less worn than that in Monson’s study, which was based on dry skulls with apparent dental attrition. The level of attrition might be one reason for the differences in Monson’s values in most subsequent studies. In addition, Ferrario et al. [9] reported a three-dimensional occlusal curvature in adolescents (12–14 years of age) of approximately 80 mm, which was smaller than the approximately 101 mm reported in adults (19–22 years of age). Ferrario et al. suggested that age significantly affects the radius of occlusal curvature. Some studies have shown that the intermolar arch width continues to decrease in adulthood [18, 19] and the buccolingual inclination of the mandibular molars varies among different age groups [20, 21]. These dentoalveolar changes indicated long-term alterations of natural dentition, which must be considered when oral rehabilitation or orthodontic treatment is performed for patients of different ages. Therefore, the results of this study may apply only to young adults.

The mean radius of 51 females included in the present study was 79.60 ± 14.13 mm. Actually, this study also collected 7 males and calculated their radii of Monson’s spheres. The individual Monson’s sphere radii in these seven males were 57.12 mm, 80.60 mm, 97.54 mm, 99.37 mm, 102.13 mm, 126.88 mm, and 148.16 mm, respectively. It seems that the male Monson’s sphere was larger than that of the female. However, limited by the sample size of males, this study did not perform a statistical comparison between the sexes. There are some controversies regarding whether occlusal curves vary between sexes. Some studies on the Curve of Spee, which represents the occlusal curve in the sagittal plane, showed that its depth was not affected by gender [22–24]. However, these studies ignored the transverse differences in dentition, which might affect the occlusal curve in three-dimensional space. The transverse dimension of the dental arch is smaller in females [25, 26]. Kagaya et al. [10]. and Nam et al. [11]. reported that the Monson’s sphere radius was significantly smaller in females than that in males, indicating the potential presence of sexual dimorphism of the occlusal curve in three-dimensional space. Thus, sexual dimorphism of the occlusal curve should be considered when planning oral rehabilitation or orthodontic therapy for the different sexes. The results of the present study may not be appropriate for males and a larger sample size of males is required for further investigation.

The radius of Monson’s sphere in Chinese young adult females was 79.60 ± 14.13 mm, smaller than the results in some other countries. Kagaya et al. [10]. calculated the radius of the sphere in healthy Japanese young adults. The result was not normally distributed, and the median sphere radius was 110.6 mm. The large variability in their results may have been due to the undefined overbite and overjet in their sample. Nam et al. reported a mean radius among Korean young adult females (*n* = 27) of 100.70 ± 19.73 mm based on digital models [11]. However, the authors did not define the relationships between the canines and molars or anterior overbite and overjet. Dentitions with different types of sagittal relationships differ in arch forms [27], which may affect Monson’s sphere. The present study included participants with individual normal occlusion and critically restricted the inclusion criteria to minimize their impact on the results. However, skeletal variations still exist even in normal occlusion, and dentoalveolar compensation appears according to the skeletal patterns [28, 29]. Monson’s theory suggests that the features of the occlusal sphere are related to the craniofacial structure. Monson thought that the sphere center was located at the glabella point and that the sphere passed bilaterally through the centers of the condyles. Recently, Casazza et al. [30] proposed a regression formula to allow individualized estimation of the radius of a Spee curve based on lateral cephalograms. Their study demonstrated the mathematical relationships between the occlusal curves and craniofacial structures. Considering the craniofacial differences between Chinese and Caucasians [7], the present study provides individualized information on the three-dimensional occlusal curves in Chinese young adult females. Apart from the Monson’s sphere radius in the Chinese population, further information regarding the relationship between craniofacial structures and occlusal curves remains to be explored based on digital models combined with CBCT, which also reflects the limitations of this study.

As shown in Table 3, the mean deviation of the selected points to their relative spheres was 0.38 ± 0.30 mm, with maximum deviations outside and inside the sphere of 0.93 ± 0.25 mm and 0.95 ± 0.30 mm, respectively. These deviations indicated that ideal dentition with perfect occlusion conforming to Monson’s sphere may not exist in nature. Although the deviation seemed too large to allow precise occlusal adjustment in the clinical setting, the information on the sphere obtained in this study can be used as a instruction for tooth alignment and cusp location.

Regarding the mean deviation of the selected points presented in Table 4; Fig. 2, most of the lingual cusps deviated more than the buccal cusps in the posterior teeth, which might have resulted from the lingual inclination of the crown [31]. While this inclination, described as torque in orthodontics, has been extensively studied, the deviation of the cusps has not yet been explored. Combined with digital software, the deviations found in the present study can be used as a new method to instruct tooth alignment in orthodontics. Figure 2 also shows that the most deviated point was the mesiolingual cusp of the first permanent molar, and that the distance between the buccal and lingual cusps of the first permanent molar was also the most apparent. The mesiolingual cusp of the first permanent molar is anatomically higher than the mesiobuccal cusp [32]. Besides, as the main functional cusp bearing occlusal force, the buccal cusps of the first permanent teeth may be much more worn than other teeth. These factors may induce an apparent distance between the buccal and lingual cusps of the first permanent molar, as shown in Fig. 2.

As shown in Table 5; Fig. 3, the most deviated points outside the spheres were located mainly at the edges of the first incisors, the buccal cusps of the first permanent molars and the lingual cusps of the second permanent molars. The most deviated points inside the sphere were mainly at the lingual cusps of the first permanent molars. Three reasons may explain these types of distribution. First, the lingual inclinations of the clinical crowns from anterior to posterior progressively increase in the mandible [31, 33], which results in the main distribution of the lingual cusps of the second permanent molars outside Monson’s spheres. Secondly, the morphological differences in the mesiobuccal and mesiolingual cusps of the first permanent molars, as mentioned above, may result in a higher distribution of most deviated points outside and inside the spheres at the buccal and lingual cusps, respectively. Besides, the point at incisal edge is one of the highest points and most anterior point of the curve of Spee, which might result in the deviation and distribution showing in Figs. 2 and 3. Therefore, the deviation of selected points at the incisal edges and buccal and lingual cusps of the molars required increased attention when it comes to tooth alignment during orthodontic therapy or occlusal reconstruction. When 3D software is used to align teeth virtually, the Monson’s sphere could be used as a kind of template and the deviations of all the points could help to adjust the locations of different incisal edges and cusps. Further exploration remains to be done for that application.

This study investigated the characteristics of Monson’s sphere in Chinese young adult females with individual normal occlusions. The results provided further information on the three-dimensional occlusal curve for orthodontic treatment and oral rehabilitation in Chinese young adult females. However, due to the limited sample size, further investigation is needed. In addition, it is necessary to collect craniofacial data from CBCT for integration with existing digital models for in-depth research.

## Conclusion

The radius of Monson’s sphere in Chinese young adult females was smaller than the classic 4-inch value suggested by Monson. We observed some deviation from all the selected points to their fitting sphere surfaces, and the points with the greatest deviation were distributed primarily in the molar region. Deviation from each cusp or incisal edge to the surface of the sphere, as well as the patient’s race and sex should be considered when occlusal reconstruction is planned during prosthodontic or orthodontic treatment. Further researches are needed to investigate whether the occlusal sphere of men is larger than that of women. Craniofacial data from CBCT must be collected for in-depth research on the relationship between Monson’s sphere and craniofacial structure.

## Data Availability

The datasets used and/or analyzed during the current study are not publicly available but are available from the corresponding author on reasonable request.

## Abbreviations

CBCT: cone-beam computed tomography
ICC: intraclass correlation coefficient
